# Corrosion Behaviors of Ni80A Alloy Valve in Marine Engine Within Ammonia-Rich Environment

**DOI:** 10.3390/ma18133006

**Published:** 2025-06-25

**Authors:** Ying-ying Liu, Guo-zheng Quan, Yan-ze Yu, Wen-jing Ran, Wei Xiong

**Affiliations:** 1 Chongqing Key Laboratory of Advanced Mold Intelligent Manufacturing, School of Material Science and Engineering, Chongqing University, Chongqing 400044, China; 2 Jiangsu Yutaida Industrial Technology Co., Ltd., Taizhou 225300, China; 3 Huan Ding Intelligent Technology (Suzhou) Co., Ltd., Suzhou 215000, China; 4 Key Laboratory of Advanced Reactor Engineering and Safety of Ministry of Education, Collaborative Innovation Center of Advanced Nuclear Energy Technology, Institute of Nuclear and New Energy Technology, Tsinghua University, Beijing 100084, China

**Keywords:** ammonia-fueled engine, valve, computational fluid dynamics simulation, ammonia corrosion

## Abstract

Ammonia fuel is regarded as a promising zero-carbon alternative to diesel in next-generation marine engines. However, the high-temperature ammonia-rich environment poses significant corrosion challenges to hot-end components such as valves. This study investigates the corrosion behavior of Ni80A alloy marine valves under the coupled effects of a high temperature and ammonia atmosphere. Using computational fluid dynamics (CFD), the service temperature of the valve and the ammonia concentration distribution inside the engine cylinder were identified. High-temperature corrosion experiments were conducted with a custom-designed setup. Results show that corrosion kinetics accelerated markedly with temperature: the initial corrosion rate at 800 °C was four times that at 500 °C, and the maximum corrosion layer thickness reached 37 μm—double that at lower temperatures. Microstructural analysis revealed a transition from a dense, defect-free corrosion layer at 500 °C to a non-uniform layer with coarse CrN particles and aggregated nitrides at 800 °C. Notably, surface hardness increased at both temperatures, peaking at 590 HV at 500 °C, while matrix hardness at 800 °C declined due to γ′ phase coarsening and grain growth. This work provides detailed insight into the temperature-dependent ammonia corrosion mechanisms of marine Ni-based alloy valves, offering essential data for material design and durability assessment in ammonia-fueled marine engines.

## 1. Introduction

The ambitious carbon neutrality targets set by the International Maritime Organization (IMO) have accelerated the adoption of zero-carbon fuels in the global shipping industry [1,2]. Among various candidates, ammonia (NH_3_) has emerged as a promising alternative to diesel due to its carbon-free emissions and ease of storage and transport [3,4]. More importantly, ammonia fuel can run in current compression ignition (CI) engines with only minor changes in the fuel injection system. This compatibility makes it a key option in the decarbonization plan by the IMO [5,6]. However, ammonia-fueled engines operate under more aggressive conditions than conventional diesel engines. Within the combustion chamber, reactive species such as NH_2_, NH, H, and N—originating from ammonia decomposition, unburned fuel, and nitrogen oxides (NO_x_)—form a complex corrosive environment. This promotes several degradation mechanisms, including nitridation, oxidation, and hydrogen embrittlement, especially on hot-end components such as the cylinder liner and valves [7,8,9]. These effects are further intensified by frequent thermal cycling and high operating temperatures. Despite the severity of these conditions, studies on the corrosion behavior of critical components in ammonia-fueled marine engines under realistic service conditions remain limited. Among these components, the gas valve is critical, as it plays a decisive role in engine durability and reliability. Therefore, it warrants a focused investigation.

Ammonia’s corrosiveness primarily results from its metal-complexing nature, which facilitates the formation of metal–ammonia complexes. These complexes tend to lower the electrochemical potential of metallic materials, enhancing their reactivity. Most existing studies have focused on Fe- and Cu-based alloys. For example, Sun et al. [10] studied the corrosion behavior of carbon steel in an ammonia solution, finding that the corrosion occurs by forming ammoniated Fe(II) complexes. Similarly, Wei et al. [11] showed that an N_2_-NH_3_ mixture can convert Fe_2_O_3_ to Fe_3_O_4_. Shi et al. [12] investigated the ammonia corrosion mechanisms in nanocrystalline and coarse-grain Cu, demonstrating that the compact passive film on coarse-grain Cu provides adequate protection. Salcedo et al. [13] built a molecular model to study ammonia diffusion in an inhibitor film on copper, revealing that lubricants containing sulfonate additives offer superior protection for copper sensors exposed to ammonia.

In contrast, limited attention has been paid to the ammonia corrosion behavior of Ni-based alloys, particularly under marine conditions. This gap is likely due to the relatively recent emergence of ammonia as a marine fuel and the complexity of simulating such corrosive environments, which has constrained experimental investigation. Ni-based alloys such as Inconel and Hastelloy are well known for their high-temperature corrosion resistance. Singh et al. [14] analyzed the failure of Alloy 625 ammonia cracker tube stub ends after 47,000 h, identifying premature M23C6 carbide formation at grain boundaries caused by Ti consumption in nitrides, freeing carbon for carbide precipitation. Chakravartty et al. [15] studied Alloy 625 ammonia cracker tubes after 100,000 h at 600 °C, finding precipitation-driven strength gains but reduced ductility/toughness (varying by location), with elevated-temperature tests showing softening and solution annealing restoring original properties. Ghara et al. [16] exposed Inconel 600 specimens to ammonia flow at 400 °C to 600 °C in different furnace positions, identifying severe surface damage and elucidating how specimen position affects degradation extent and damage mechanisms. These studies exhibit common limitations. They are confined mainly to static or steady-state conditions typical of industrial ammonia processing. Furthermore, the extended exposure durations—often exceeding tens of thousands of hours—are not readily applicable to accelerated testing protocols required for material selection and engine design.

Moreover, several studies have noted that ammonia readily decomposes at elevated temperatures, especially in the presence of catalytic metals like nickel and iron [17,18,19]. This process leads to the formation of atomic nitrogen and hydrogen, which can diffuse into metal substrates, potentially causing nitriding and hydrogen embrittlement. Shen et al. [20] found that the addition of ammonia in AISI 5140 steel promotes the formation of Fe_3_O_4_ in the oxide layer, which can improve both the corrosion resistance and wear resistance of the substrate material. Thierry et al. [21] studied the atmospheric corrosion of hot-dip galvanized and zinc alloy-coated steel in environments containing ammonia and ammonium chloride—they observed their effects on the corrosion mechanisms. Wu et al. [22] investigated the corrosion and erosion coupling behavior of a fluidized bed boiler in ammonia gas and found that the high ammonia concentration accelerates the wear of specimens. Despite these efforts, existing studies often fail to replicate the combined thermal, mechanical, and chemical stresses present in real marine engines, limiting their relevance to component design and durability assessment. Experimental replication of these conditions is particularly challenging due to the high-temperature and high-pressure environment inside the engine cylinder, which can rapidly damage test equipment.

**Figure 1:**
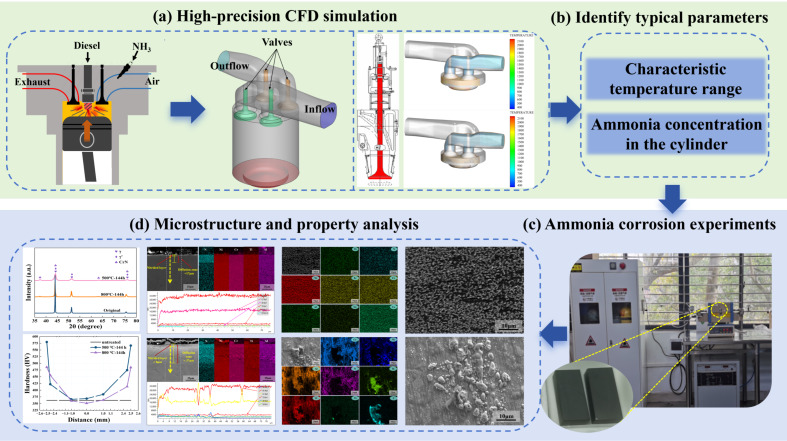
Studying framework for ammonia corrosion behaviors of marine valve.

To address this knowledge gap, the present study investigates the high-temperature ammonia corrosion behavior of Ni80A alloy, a material commonly used in marine valves. It is hypothesized that the corrosion resistance of Ni80A is significantly influenced by elevated temperatures and ammonia concentrations typical of ammonia-fueled engines. A combined approach involving computational fluid dynamics (CFD) simulation and high-temperature corrosion testing was employed (Figure 1). A high-fidelity CFD model of a marine ammonia engine was developed to simulate the full engine cycle, including intake, compression, combustion, and exhaust strokes. This provided key data on ammonia concentration and surface temperature at the gas valve. Based on the simulation results, two representative temperatures (500 °C and 800 °C) were selected to represent the range where ammonia remains stable and chemically active. Corrosion tests were conducted using a custom-built high-temperature experimental setup, and the effects of ammonia on Ni80A were evaluated through microstructural analysis and mechanical testing. The findings of this study aim to provide practical guidance for material selection and design of critical components in next-generation ammonia-powered marine engines.

## 2. Experimental Methods

### 2.1. Service Temperature Identification of Valve

**Figure 2:**
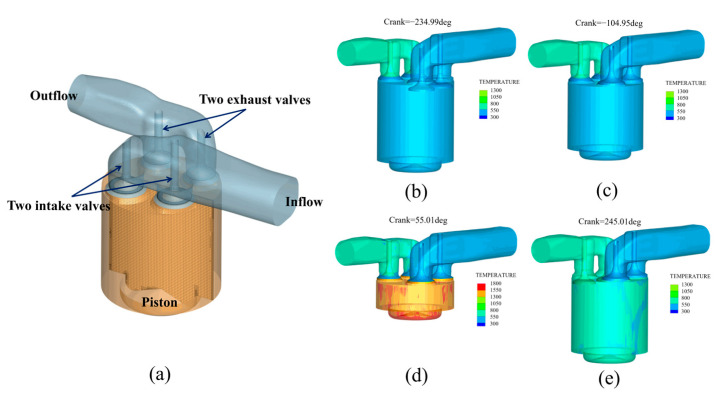
Flow field model in the cylinder of a four-stroke marine engine and the simulation results of temperature distribution at different strokes: (**a**) mesh model; (**b**) intake stroke; (**c**) compression stroke; (**d**) power stroke; (**e**) exhaust stroke.

The temperature plays a crucial role in influencing the corrosion behaviors of Ni80A valves in marine engines within an ammonia-rich environment. In this work, the service temperature of the valve and the ammonia concentration inside the engine cylinder were identified through computational fluid dynamics (CFD) simulation using CONVERGE 3 (Convergent Science Inc., Madison, WI, USA). A three-dimensional transient combustion simulation model for an ammonia–diesel dual-fuel system was developed based on a four-stroke marine engine. Figure 2a displays the flow field model in the cylinder of a four-stroke marine engine, and Figure 2b–e exhibits the simulation results of temperature distribution at different strokes. In the CFD model, the geometric features of the in-cylinder flow field include intake ports, combustion chamber, valve assembly, and exhaust ports. Dynamic layering mesh technology was used to simulate the periodic motion of the valves and pistons, with a global maximum mesh size of 1 mm. A triple-layer mesh refinement strategy was applied to the sealing areas of the valves to resolve the boundary layer behavior near the valve surfaces. Furthermore, an adaptive mesh refinement (AMR) method based on local vorticity gradients was implemented to address rapid flow field variations during the valve opening and closing processes. This approach enabled the generation of fine meshes with a size of 0.1 mm around the valve sealing regions, ensuring accurate capture of the spatiotemporal evolution of NH_3_ concentration without the need for manually constructing multiple grid levels. The simulation boundary conditions were defined based on typical marine engine operating data. Specifically, the intake port pressure and temperature were set to 1.83 bar and 309 K, respectively, while the exhaust port pressure and temperature were set to 1.01 bar and 800 K. The initial in-cylinder conditions were initialized according to compression stroke data corresponding to 75% engine load and 1600 rpm. Although a traditional mesh independence study was not performed due to the adaptive meshing strategy, a convergence check was carried out by adjusting mesh refinement and time-step control parameters. The variations in key results—such as in peak in-cylinder pressure and heat release rate—remained within 6.0%, confirming the mesh-independent behavior and numerical stability of the solution under the present settings. The chemical kinetics were calculated based on a combined n-heptane/NH_3_ reaction mechanism, which includes 112 species and 786 elementary reactions. The mechanism focuses on the ammonia decomposition pathway (NH_3_ → NH_2_ → NH) and its interaction with diesel combustion [23]. Fuel spray breakup was modeled using the KH-RT model, while the evaporation of fuels was described using the Frossling-corrected model [24,25,26]. The detailed models used in the CFD simulation, along with their justifications, are summarized in Table 1.

**Figure 3:**
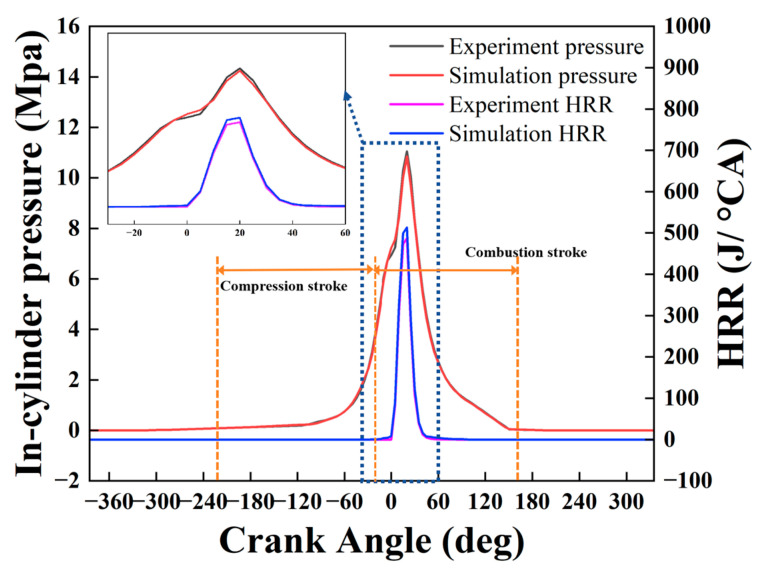
Comparison of in-cylinder pressure and heat release rate (HRR) between simulation and experimental results. The close agreement validates the simulation model and confirms its capability to accurately capture combustion characteristics under dual-fuel conditions.

In-cylinder pressure and heat release rate (HRR) were used as key indicators to evaluate the accuracy of the simulation model. These validation metrics are consistent with those widely adopted in similar studies [27,28,29]. Figure 3 presents the comparison between simulated and experimental results for in-cylinder pressure and HRR. As shown, both the timing and magnitude of the peak pressure, as well as the HRR trends, agree well with the experimental data. The absolute relative error (ARE) of the in-cylinder pressure remains within 5.1%, while that of HRR is within 6.0%. These results demonstrate that the developed CFD model for the ammonia–diesel dual-fuel marine engine can reliably capture the key combustion characteristics.

**Figure 4:**
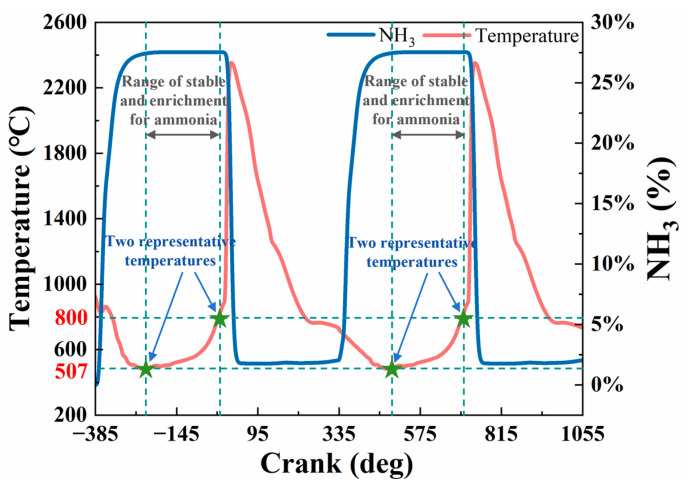
Variations in in-cylinder temperature and NH_3_ concentration with crank angle over two engine cycles. NH_3_ concentration peaks during the compression stroke, while temperature rapidly increases in the early power stroke, reaching 2400 °C and triggering NH_3_ decomposition. These trends highlight the strong thermo-chemical coupling in dual-fuel combustion and inform the selection of key test temperatures for corrosion analysis.

To elucidate the dynamic evolution of the in-cylinder flow field in a four-stroke marine engine, temperature and NH_3_ concentration profiles were extracted from CFD simulation results and plotted against the crank angle over two engine cycles, as shown in Figure 4. During the compression stroke, the NH_3_ concentration consistently peaks at a molar fraction of approximately 30% while the corresponding temperature ranges from 507 °C to 800 °C. In the early power stroke, the temperature rises sharply, reaching a maximum of 2400 °C, which triggers NH_3_ ignition and subsequent decomposition, resulting in a rapid decline in its concentration. These results demonstrate the transient thermo-chemical coupling behavior inherent in dual-fuel combustion.

Based on the simulation results, the ammonia-rich region is primarily located during the compression stroke, with a typical temperature range of 500–800 °C. This temperature range was derived from transient CFD simulations, which account for the time-dependent evolution of thermal and chemical conditions under realistic engine operating cycles. This range is considered critical for corrosion evaluation, as it reflects the most relevant thermo-chemical environment in ammonia-containing conditions—conditions under which material degradation is most likely to occur. Considering both the representativeness of this temperature range and the practical feasibility of long-duration corrosion experiments, two fixed temperatures—500 °C and 800 °C—were selected as representative test conditions.

### 2.2. Ammonia Corrosion Experiments

**Figure 5:**
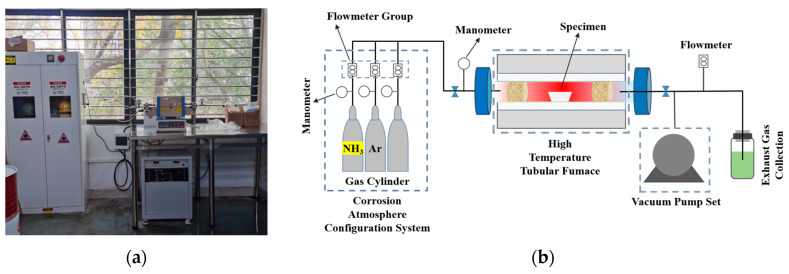
The practical picture (**a**) and schematic diagram (**b**) of the experiment device.

Based on the identified service temperatures of the valve (500 °C and 800 °C), accelerated ammonia corrosion experiments were conducted using Ni80A alloy samples. The tests were conducted using a self-developed experimental setup consisting of an OTF-1200× tubular vacuum furnace, a vacuum pump, a gas cylinder cabinet, and a gas circuit system. The device enabled precise control of gas composition and pressure, allowing for the simulation of high-temperature environments under various atmospheres. Both the actual photograph and the schematic diagram of the setup are shown in Figure 5. The chemical composition of Ni80A alloy samples is shown in Table 2, with an initial hardness of 365 HV. Prior to testing, samples were pre-treated by aging treatment at 720 °C for 8 h, followed by air-cooling to promote γ′ phase precipitation. The specimens (20 × 10 × 5 mm^3^) were mechanically ground (80–3000 mesh) and polished using 0.05 μm diamond suspension to a final surface roughness of Ra < 0.02 μm. They were then ultrasonically cleaned in an acetone–ethanol solution and stored in dry nitrogen to minimize surface oxidation. For each test condition (500 °C and 800 °C), three parallel samples were used to ensure repeatability, and the results are presented as average values.

**Figure 6:**
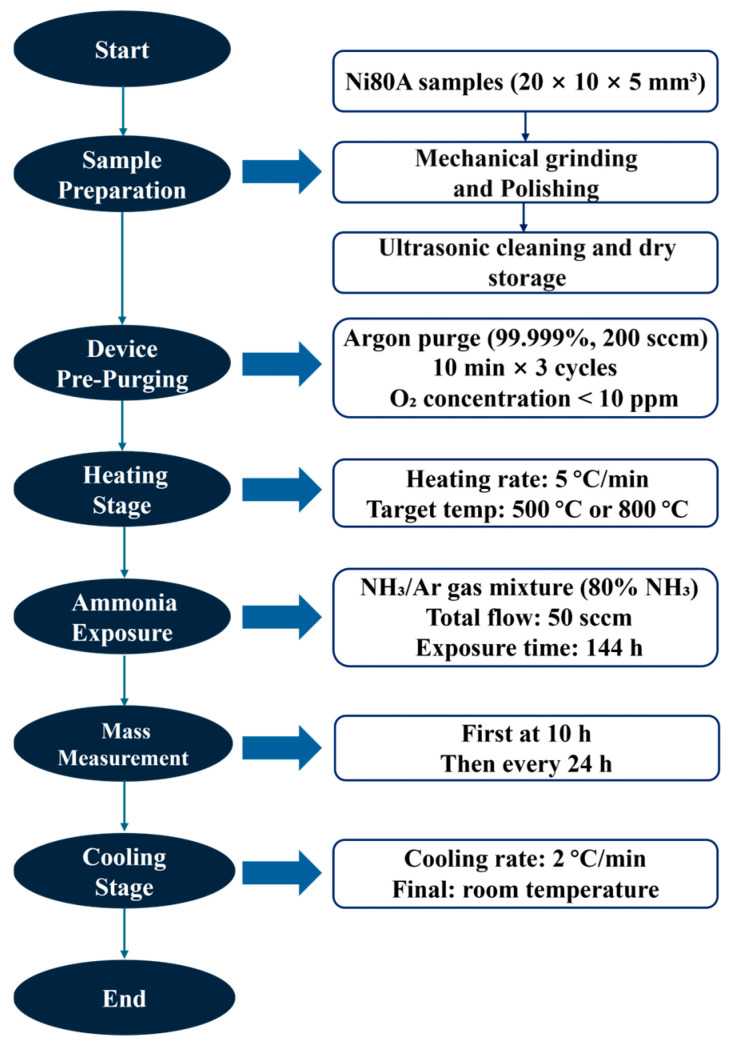
Flowchart of the ammonia corrosion experiment.

Figure 6 presents a flow chart of the entire experimental procedure. Firstly, high-purity argon (99.999%) was flushed into the quartz tube at 200 sccm for 10 min × 3 cycles to reduce the oxygen content to below 10 ppm. Then, the temperature was increased at a rate of 5 °C/min until it reached the target temperature (500 °C or 800 °C), which was selected based on CFD-predicted valve service temperatures under ammonia-rich conditions. Subsequently, the gas atmosphere was switched to an NH_3_/Ar mixture (80 vol.% NH_3_, total flow rate: 50 sccm), with these parameters inspired by published research [30]. The exposure duration of 144 h was selected to ensure adequate corrosion development under moderate ammonia and temperature conditions. This time frame provides a balance between generating observable corrosion phenomena and maintaining practical feasibility for laboratory testing. The mass change was first recorded after 10 h of exposure and subsequently measured every 24 h. Each data point was obtained by conducting three repeated measurements, and the average value was used as the final result. After the experiment, the samples were cooled at 2 °C/min to room temperature to prevent cracking of the corrosion layer due to thermal stress.

### 2.3. Characterization and Testing Methods

Through corrosion kinetic curves, microstructural morphology, and the microhardness of the corrosion layer, the ammonia corrosion behaviors of the Ni80A alloy were analyzed. Herein, the weight gain method was employed to evaluate the corrosion rate of the sample by measuring the mass change per unit area. The corrosion rate was calculated using Equation (1). Corrosion kinetics curves were then fitted and plotted with corrosion time on the *x*-axis and weight gain per unit area on the *y*-axis.

(1) $$V=\left(M_{t}-M_{0}\right)/St=∆M/St$$

where *M*_0_ is the initial mass of sample; *M_t_* is the mass of sample after corrosion; ∆M is the mass variation in the sample before and after corrosion; V is the corrosion rate; *S* is the surface area exposed to the ammonia-rich environment; *t* is the corrosion time.

For microstructural characterization, scanning electron microscopy (SEM, JSM-7800F, JEOL, Tokyo, Japan) was employed to examine both the surface and cross-sectional morphologies of the corrosion layers. Prior to cross-sectional analysis, the corroded samples were sectioned perpendicular to the exposed surface using a low-speed precision cutter. The cross-sections were then mounted in epoxy resin, mechanically ground using SiC papers up to 3000 grit, and subsequently polished with 0.05 μm diamond suspension to ensure a smooth finish for imaging. The observation was conducted along the plane normal to the corroded surface to reveal the corrosion depth and layer structure clearly. For elemental composition analysis, an Oxford X-Max 80 energy-dispersive X-ray spectroscopy (EDS) system (Oxford Instruments, Abingdon, UK) was utilized to examine the diffusion gradients of nitrogen (N), chromium (Cr), and nickel (Ni) along the depth direction, allowing tracking of the chemical evolution at the corrosion front. Nitrogen diffusion depth was evaluated based on the elemental concentration profile obtained from EDS line scans using SEM instrument, with the diffusion boundary defined at the point where nitrogen concentration dropped to 5% above the baseline. Phase identification was conducted on a Bruker D8 Advance X-ray diffractometer (XRD) (Bruker, Billerica, MA, USA) using Cu Kα radiation with a scanning range of 10–90°, and a step size of 0.02° [31,32], and full-spectrum fitting was performed using Jade 9.0 software to analyze the volume fraction evolution of characteristic phases within the corrosion layer. The mechanical properties were evaluated using a Vickers microhardness tester (HV-1000Z, Shanghai Optical Instrument Factory, Shanghai, China) with a selected load of 500 g and a dwell time of 15 s. The 500 g load was selected as it offers an appropriate balance between producing sufficiently clear indentations and minimizing substrate influence, which is particularly important for capturing hardness gradients within the corrosion layer. Measurements were carried out along the depth direction of the corrosion layer, with indentations spaced approximately five μm apart to prevent mechanical interaction between adjacent impressions. Each hardness value represents the average of three independent measurements at the same depth. The standard deviation of the measurements was within ±5 HV, ensuring the statistical reliability of the results.

## 3. Results and Discussion

### 3.1. Analysis of Corrosion Kinetics

**Figure 7:**
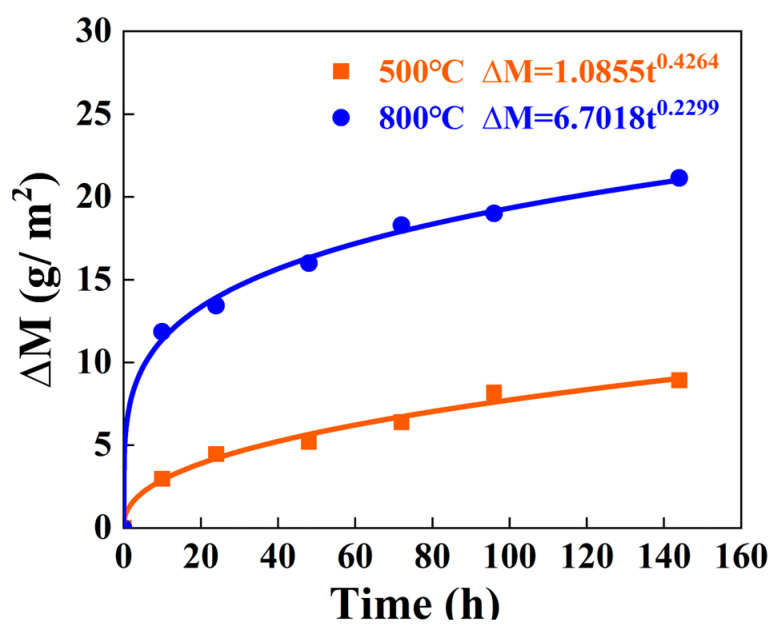
The corrosion kinetics curves of samples corroded at 500 °C and 800 °C.

According to Equation (1), the mass variations and the corrosion rates of the samples corroded at different temperatures for 10 h and 144 h are calculated, as given in Table 3. It can be seen that during the initial stage of the corrosion process (0–10 h), the corrosion rate of the samples corroded at 500 °C is 0.2975 g/(m^2^·h). In contrast, the corrosion rate of samples corroded at 800 °C exhibits a significantly higher value of 1.1857 g/(m^2^·h), approximately four times greater than that observed at 500 °C. The results indicate that elevated temperature significantly enhances the corrosion rate of the sample. As the corrosion time was extended to 144 h, the corrosion kinetics curves of the corroded samples at different temperatures are plotted in Figure 7. These curves exhibit significant parabolic characteristics. At the end of the corrosion period, the corrosion rates at 500 °C and 800 °C decrease to 0.062 g/(m^2^·h) and 0.1468 g/(m^2^·h), respectively. The corrosion rate at 800 °C is approximately 2.4 times greater than that at 500 °C, indicating the significant and persistent influence of temperature on the corrosion behaviors. The severe corrosion kinetics at elevated temperatures can be attributed to the enhanced thermodynamic driving force and the accelerated diffusion process of reactive species, thereby accelerating the corrosion process.

### 3.2. Analysis of Corrosion Behaviors

**Figure 8:**
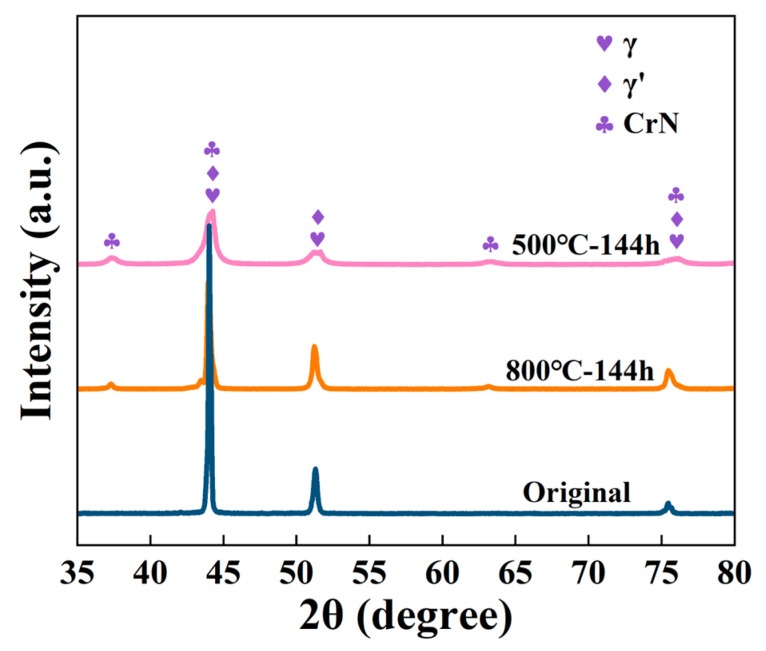
XRD patterns of the original specimen and corroded samples.

Figure 8 shows the XRD patterns of the original sample and corroded samples. It is evident that in all samples, the strongest diffraction peaks of the γ′ phase are observed to overlap with those of the γ phase to keep their coherence relationship. The intensities of γ phase diffraction peaks are found to decrease after corrosion at both temperatures when compared to the original sample. The significant difference in Figure 8 is that distinct CrN diffraction peaks are detected for the samples corroded at both 500 °C and 800 °C. It suggests that ammonia (NH_3_) is decomposed at both temperatures. The corrosion process involves complex interactions between the material and its environment. While ammonia (NH_3_) itself does not directly undergo electrochemical reactions, it decomposes at high temperatures into nitrogen (N_2_) and hydrogen (H_2_). The nitrogen atoms produced from this decomposition diffuse into the alloy surface, where they react with chromium (Cr) to form CrN. This mechanism explains the formation of CrN at both 500 °C and 800 °C. The diffraction intensity of CrN peaks slightly decreases at 800 °C compared to the sample corroded at 500 °C, indicating that CrN is less stable at higher temperatures. This could be due to the increased oxidation of chromium at 800 °C, which may lead to the decomposition of some CrN, suggesting a temperature-dependent stability of CrN in the corrosion layer. In addition, the diffraction intensity of CrN peaks slightly decreases at 800 °C compared to the sample corroded at 500 °C. This decreasing trend indicates that the content of CrN particles in the corrosion layer formed at 800 °C is lower than that at 500 °C.

**Figure 9:**
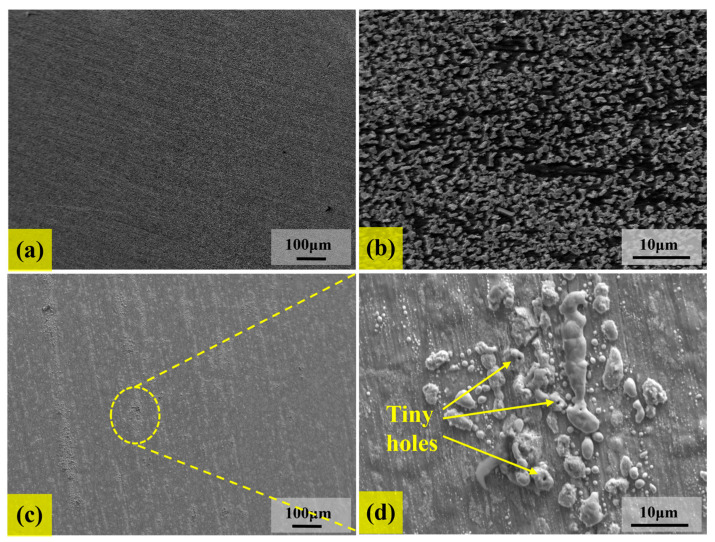
The surface morphology of the corrosion layer for specimens corroded at different temperatures: (**a**,**b**) 500 °C; (**c**,**d**) 800 °C.

**Figure 10:**
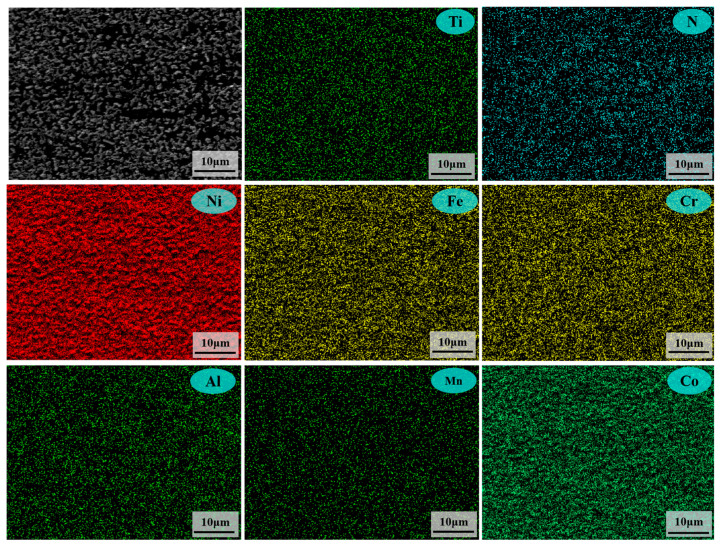
The EDS mapping of microscopic morphology after corrosion at 500 °C for 144 h.

**Figure 11:**
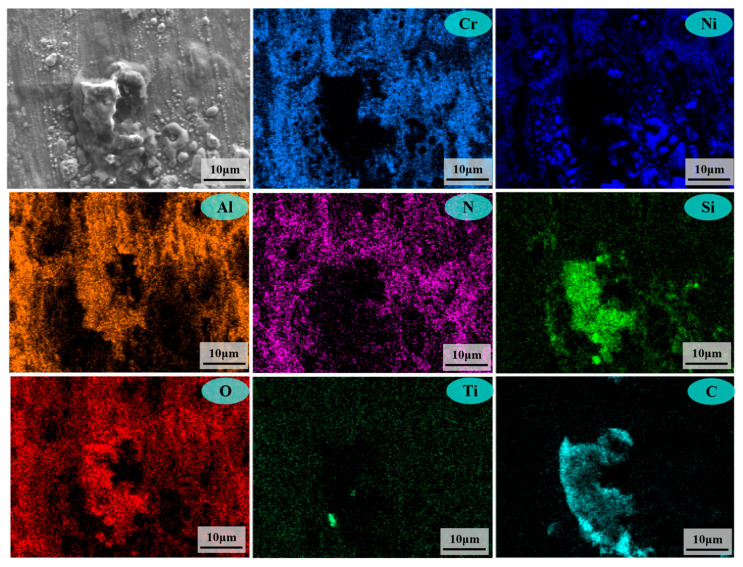
The EDS mapping of microscopic morphology after corrosion at 800 °C for 144 h.

Microstructural characterization further reveals the influence of temperature on the ammonia corrosion behaviors of samples. Figure 9 shows the microscopic morphology of the samples corroded at 500 °C and 800 °C for 144 h, respectively. The corresponding EDS mapping is shown in Figure 10 and Figure 11. Figure 9 reveals that the surface of corrosion layers is predominantly covered with nitrides. Combined with the EDS mapping in Figure 10 and Figure 11, the nitride particles at both temperatures are primarily composed of CrN, which is consistent with the XRD analysis results of the corroded samples.

At 500 °C, the surface of the corrosion layer exhibits a uniform and dense surface without apparent defects, and the consistent growth trajectory of nitride particles at this temperature is observable, as depicted in Figure 9a,b. In contrast, at 800 °C, the corrosion layer shows significant inhomogeneity, as illustrated in Figure 9c,d. The nitride particles on the surface of the corrosion layer appear relatively coarse and are locally aggregated. Furthermore, tiny pores are wrapped in the coarse nitride particles. This morphological difference mainly stems from the influence of temperature on the reaction-diffusion mechanism. At 500 °C, the diffusion of nitrogen atoms is relatively slow, resulting in a smooth nitrogen concentration gradient and isotropic growth [33]. At 800 °C, the elevated temperature accelerates the surface reaction, disrupting the dynamic equilibrium between the rapid surface reaction and the nitrogen diffusion rate, resulting in an unstable nitrogen concentration gradient and triggering local nucleation and abnormal growth behaviors [34]. Additionally, the increased temperature accelerates the decomposition of ammonia. When the concentration of nitrogen atoms is too high, nitrogen gas may be wrapped by CrN particles, resulting in the formation of pores [35,36], as shown in Figure 9d.

**Figure 12:**
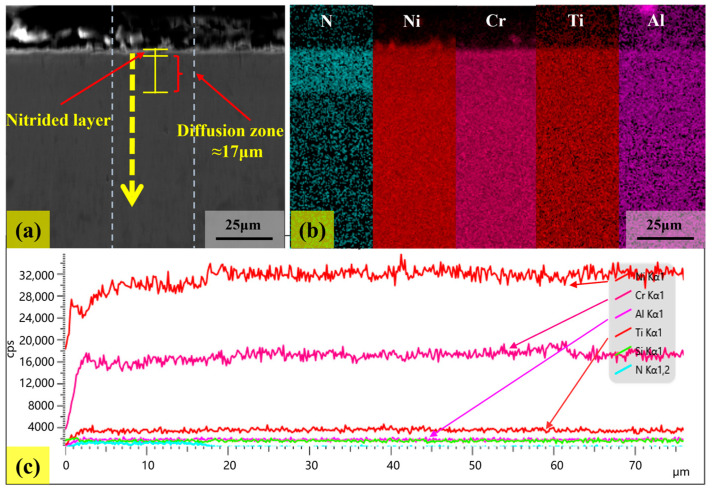
The cross-sectional morphology (**a**), surface scan (**b**), and line scan (**c**) of the sample corroded at 500 °C for 144 h.

**Figure 13:**
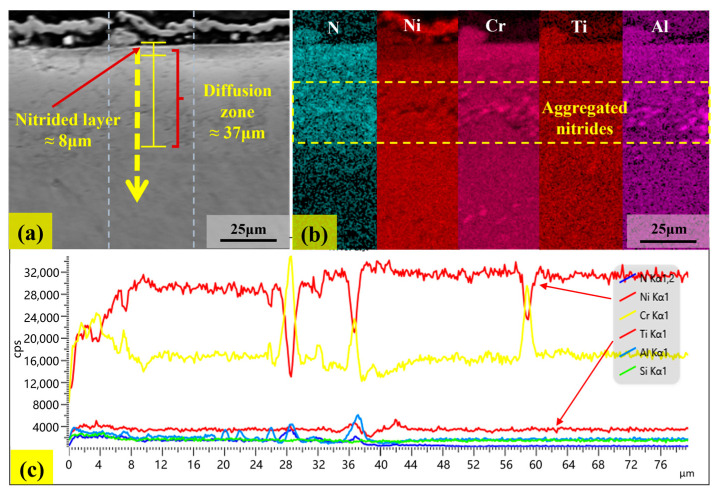
The cross-sectional morphology (**a**), surface scan (**b**), and line scan (**c**) patterns of the sample corroded at 800 °C for 144 h.

Figure 12 and Figure 13 show the cross-sectional morphology, surface scan, and line scan patterns of the samples corroded at 500 °C and 800 °C for 144 h, respectively. In Figure 12a and Figure 13a, the yellow arrows represent the line scanning direction, extending from the edge of the nitride layer to the interior of the substrate. It should be noted that the nitride layer on the surface of the corroded sample is relatively thin. In Figure 12b, it can be found that after corrosion at 500 °C, nitrogen atoms form a uniform nitrogen diffusion zone within the matrix. Combined with Figure 12c, the depth of this nitrogen diffusion zone is approximately 17 μm. The dense nitride layer effectively inhibits further nitrogen diffusion, resulting in a relatively stable nitrogen diffusion zone. In contrast, at 800 °C, the corrosion layer of the sample presents a distinct two-layer structure comprising a nitride layer and a nitrogen diffusion zone, as shown in Figure 13b. Combined with Figure 13c, the thickness of the nitride layer is approximately 8 μm, and the depth of the nitrogen diffusion zone is approximately 37 μm, which is twice the depth observed at 500 °C. In addition, a new nitride aggregation area emerged in the nitrogen diffusion zone for the sample corroded at 800 °C, as shown in Figure 13b.

The expansion of the nitrogen diffusion zone at 800 °C can be attributed to the enhanced diffusion ability of nitrogen atoms at elevated temperatures, which makes them prone to migrate into the interior of sample. When the local nitrogen concentration exceeds the solid solubility limit, the excess nitrogen atoms react with alloying elements to form nitrides and subsequently aggregate. Combined with Figure 13b,c, it can be concluded that Cr, Al, and Ti in the alloy are involved in the nitride formation process. Additionally, defects such as grain boundaries and dislocations act as rapid diffusion channels for nitrogen [37]. When nitrogen atoms migrate along these channels, they tend to accumulate in regions with high defect densities. Once the concentration reaches a critical threshold, it triggers the nucleation and aggregation of nitrides.

### 3.3. Analysis of Micro-Hardness in Corrosion Layer

**Figure 14:**
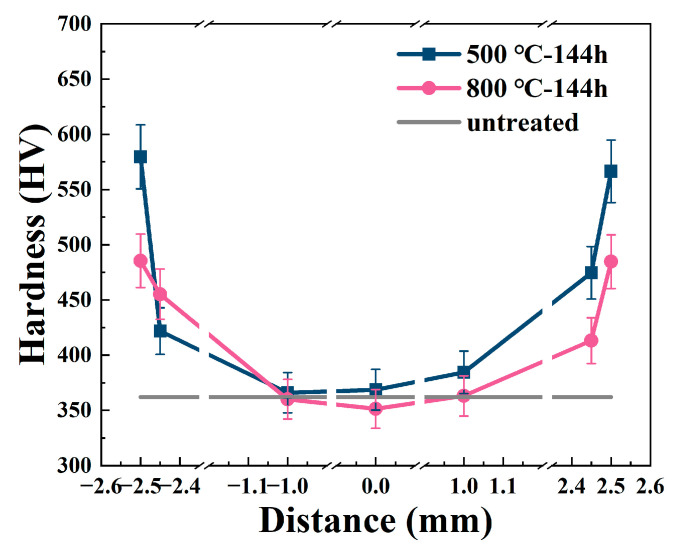
Variation in average hardness from the surface layer to the interior of the substrate.

After 144 h of corrosion at different temperatures, hardness measurements were carried out on the specimens. Surface hardness was first measured, followed by gradient hardness sampling across the cross-section, extending from one side to the other. To ensure the accuracy of the results, each hardness value was obtained by averaging three independent measurements at the same depth position. The standard deviation was also calculated and is shown as error bars in Figure 14. For both 500 °C and 800 °C corroded specimens, the hardness data were collected, as shown in Figure 14. In Figure 14, the *x*-axis spans from −2.5 mm to +2.5 mm, corresponding to the total specimen thickness of 5 mm. The positions at −2.5 mm and +2.5 mm represent the two outer surfaces, while 0 mm indicates the center of the cross-section. Negative and positive values denote locations on the left and right sides of the center, respectively. This representation facilitates a symmetric observation of hardness variations across the cross-section of the sample. In Figure 14, it can be observed that for specimens treated at both temperature levels, the hardness values exhibit a decreasing trend as the depth from the surface into the substrate increases, presenting a typical gradient distribution. The micro-hardness of the untreated specimen is approximately 365 HV. For the specimen corroded at 500 °C, the maximum hardness reaches 590 HV. Correspondingly, for the specimen corroded at 800 °C, the maximum hardness reaches 500 HV. This phenomenon indicates that during the ammonia corrosion process, the diffusion of nitrogen into the substrate enhances the surface hardness of the sample.

Notably, for the specimen corroded at 800 °C, the hardness value in the deep region of the substrate is lower than that of the untreated specimen. This is because at elevated temperatures atomic mobility intensifies. Nitrogen atoms diffuse deeply into the substrate at high temperatures and combine with alloying elements such as Cr, Al, and Ti to form nitrides (which is consistent with the EDS mapping observed in the cross-section at 800 °C), thereby weakening the solid-solution strengthening effect of the alloy. Moreover, the hardness of the substrate mainly relies on the dispersion strengthening of the γ′ phase (Ni_3_(Al, Ti)). With the prolongation of the corrosion time, some small γ′ phase particles dissolve, while large γ′ phase particles grow [38,39]. This leads to a reduction in the number of precipitated phases and an increase in the inter-particle spacing, significantly diminishing the strengthening effect. Simultaneously, the increase in temperature promotes rapid grain growth, resulting in grain coarsening and a decline in the grain-boundary strengthening effect.

In Figure 14, the hardness of the nitrided layer at 500 °C is higher than that at 800 °C. At 500 °C, the diffusion rate of nitrogen atoms is appropriate, enabling the formation of a nitrided layer with uniform thickness and dense structure. At this time, the nitrides are uniformly distributed in the form of fine particles without aggregation or coarsening. In contrast, at 800 °C, the diffusion rate of nitrogen atoms accelerates significantly. This results in an increase in the thickness of the nitrided layer. At high temperatures, the nitride particles coarsen and aggregate, forming a non-uniform distribution within the substrate, thus reducing the strengthening effect. Moreover, an excessive amount of nitrogen atoms penetrates the substrate, disrupting the structure of the γ′ phase and weakening its strengthening effect, thereby causing a decrease in the substrate strength. Additionally, high temperatures facilitate grain coarsening in the alloy, weakening the grain-boundary strengthening effect and further reducing the hardness.

## 4. Conclusions

(1) A transient combustion simulation model for an ammonia–diesel dual-fuel system was developed, and the dynamic evolution of the flow field in the cylinder of a four-stroke marine engine was revealed. Temperature and ammonia concentration distributions in the engine cylinder were identified from the CFD simulation. The service temperature range of the valve in an ammonia-rich environment was identified as 507~800 °C for the marine engine studied in this work.

(2) The corrosion behaviors of Ni80A samples corroded at 500 °C and 800 °C were revealed. The corrosion kinetics curves indicated that elevated temperature significantly enhances the corrosion rate. At 500 °C, the surface layer of the corrosion layer is composed of uniform and dense CrN, and the depth of the nitrogen diffusion zone is 17 μm. At 800 °C, the corrosion layer presents a non-uniform structure with coarsened and aggregated nitrides, and the nitrogen diffusion area reaches 37 μm.

(3) The variations in micro-hardness in the corrosion layer of the samples were uncovered. After corrosion at 500 °C and 800 °C, the surface hardness of the samples is improved, and the hardness values decrease along the depth from the surface into the substrate. The surface hardness of samples corroded at 500 °C is significantly higher than that at 800 °C, while the hardness of the matrix at 800 °C decreases due to the coarsening of the γ′ phase and grain growth.

(4) Based on the results, it is recommended that valve operating temperatures be controlled below 800 °C to reduce corrosion and maintain mechanical properties. However, this study is limited by the small number of temperature points, the absence of high-resolution characterization, and the qualitative nature of EDS and XRD analyses. Future research should incorporate broader temperature ranges and advanced techniques, such as Rietveld refinement and quantitative elemental mapping, to achieve a more comprehensive understanding of the corrosion mechanisms.

## Figures and Tables

**Table 1:** The detailed models used in the CFD simulation.

Model Name	Sub-Models and Justification
Turbulence model	RNGκ-ε model—robust for capturing turbulent kinetic energy dissipation in internal combustion engines.
Spray breakup model	KH-RT model—simulates both primary and secondary breakup accurately for high-pressure diesel sprays.
Spray/wall interaction model	Wall film model/wall film–O’Rourke model—captures droplet–wall interaction and film dynamics under wall impingement conditions.
Droplet collision model	NTC collision model—accounts for non-linear droplet collision effects, improving accuracy in dense spray regions.
Wall heat transfer model	Han and Reitz model—incorporates film cooling and boiling effects, providing realistic heat transfer prediction at the wall.
Combustion model	SAGE model—a detailed chemical kinetics solver suitable for multi-component fuel combustion in diesel engines.
NO_x_ Emission Model	Extended Zeldovich NO_x_ model—widely validated for thermal NO_x_ formation in high-temperature combustion environments.
Evaporation	Frossling model—considers convective heat and mass transfer effects, appropriate for fuel droplet evaporation under transient engine conditions.

**Table 2:** Chemical composition of Ni80A alloy (wt.%).

Element	Cr	Ti	Al	Fe	Mn	Si	C	Ni
Content	20.88	2.02	1.48	1.15	0.52	0.61	0.063	Balance

**Table 3:** Mass variations and corrosion rates of the samples corroded at 500 °C and 800 °C.

Temperature	*S* (m^2^)	*M*_0_ (g)	*M*_10h_ (g)	*M*_144h_ (g)	*V*_10h_ g/(m^2^·h)	*V*_144h_ g/(m^2^·h)
500 °C	0.0007	7.6092	7.6112	7.6152	0.2975	0.0620
800 °C	0.0007	7.4735	7.4818	7.4883	1.1857	0.1468

## Data Availability

The original contributions presented in this study are included in the article. Further inquiries can be directed to the corresponding authors.
